# Intraoperative Seizures in Glioma Surgery: Is It Really Only an Intraoperative Issue?

**DOI:** 10.3390/cancers17091478

**Published:** 2025-04-27

**Authors:** Giada Pauletto, Annacarmen Nilo, Christian Lettieri, Mariarosaria Valente, Marco Vindigni, Miran Skrap, Tamara Ius, Lorenzo Verriello

**Affiliations:** 1 Neurology Unit, Head-Neck and Neurosciences Department, Santa Maria Della Misericordia University Hospital, 33100 Udine, Italy; giada.pauletto@asufc.sanita.fvg.it (G.P.); lorenzo.verriello@asufc.sanita.fvg.it (L.V.); 2 Clinical Neurology Unit, Head-Neck and Neurosciences Department, Santa Maria Della Misericordia University Hospital, 33100 Udine, Italy; christian.lettieri@asufc.sanita.fvg.it (C.L.); mariarosaria.valente@uniud.it (M.V.); 3 Department of Medicine (DMED), University of Udine, 33100 Udine, Italy; 4 Neurosurgery Unit, Head-Neck and Neurosciences Department, Santa Maria Della Misericordia University Hospital, 33100 Udine, Italy; marco.vindigni@asufc.sanita.fvg.it (M.V.); miran.skrap@gmail.com (M.S.); 5 Academic Neurosurgery, Department of Neurosurgery, University of Padova, 35121 Padova, Italy; tamara.ius@unipd.it

**Keywords:** low-grade glioma, intraoperative seizures, tumor-related epilepsy, post-operative seizure outcome, clinical outcome

## Abstract

**Background/Objectives**: Intraoperative seizures (IOS) occur in approximately 2.9–54.3% of glioma surgeries and are often considered provoked events. Notwithstanding, their impact on seizure control and clinical outcomes remains unclear. Our aim is to assess the potential role of IOS on short- and long-term seizure and clinical outcomes in patients with tumor-related epilepsy (TRE). **Methods**: This is a retrospective, single-center study of 154 patients with glioma and TRE who underwent surgery. Seizure and clinical outcomes were evaluated considering the Engel classification, the Karnofsky Performance Status (KPS) scale, and the modified Rankin Scale (mRS), at 12 and 24 months of follow-up. **Results**: IOS occurred in 40 patients (26%), mostly during awake surgery, without changing the anesthesiologic protocol. Early post-operative seizures (POS) were observed in 28 patients (18.5%). There was no significant difference in the risk of developing POS between patients with and without IOS (*p* = 0.782). IOS did not impact seizure or clinical outcomes at 12 (*p* = 0.403; *p* = 0.748; *p* = 0.363) and 24 months (*p* = 0.488; *p* = 0.975; *p* = 0.538). In contrast, early POS were associated with worse seizure control at 12 months (*p* = 0.005) but not at 24 months (*p* = 0.102). No significant correlations were found between early POS and clinical outcomes at 1 (*p* = 0.324; *p* = 0.168) and 2 years (*p* = 0.920; *p* = 0.165). **Conclusions**: IOS appear to be disruptive only intraoperatively, not influencing short- and long-term seizure control and clinical outcomes. In contrast, early POS have a higher impact, particularly during the first post-operative year.

## Simple Summary

Intraoperative seizures (IOS) occur frequently during glioma surgery, but their impact on long-term seizure control and clinical outcomes remains unclear. This study retrospectively analyzed 154 patients with tumor-related epilepsy (TRE) who underwent glioma surgery, assessing seizure and clinical outcomes at 12 and 24 months. IOS were observed in 26% of patients, mostly during awake surgery, without changing anesthetic management. Early post-operative seizures (POS) occurred in 18.5% of patients. IOS did not significantly affect seizure control or clinical outcomes at both time points. However, early POS were associated with worse seizure control at 12 months, though this association weakened by 24 months. No significant correlation was found between early POS and clinical outcome. These findings suggest that while IOS may be disruptive intraoperatively, they do not influence long-term prognosis. In contrast, early POS have a more pronounced impact, particularly in the first post-operative year.

## 1. Introduction

Intraoperative seizures (IOS) represent a complication during surgery of diffuse low-grade gliomas (DLGGs), particularly in cases of awake craniotomy, history of tumor-related epilepsy (TRE), and cortical mapping [1,2]. In the literature, the incidence of IOS varies widely, ranging from 2.9 to 54.3%, depending on methodology, patient population, surgical approach, intra-operative mapping characteristics, and whether electroencephalographic techniques were employed to detect electrical seizures and/or seizures with non-motor semeiology [3].

IOS have always been considered provoked seizures, with an impact only on intraoperative management. Several studies analyzed risk factors for IOS [2] and the harmful intraoperative consequences, mainly represented by the evolution into tonic–clonic seizures, loss of patients’ cooperation, need for propofol or benzodiazepines drip, alterations in anesthetic parameters (O2 saturation, heart rate, blood pressure) and the necessity of varying levels of sedation, potentially requiring intubation, thereby undermining the benefits of awake procedures [4].

Notwithstanding, less is known about the effect of IOS on post-operative seizure and clinical outcomes [1]. This may be attributed to various factors, such as fragmented management of patients with brain tumors and epilepsy, as well as the underestimation of IOS themselves across surgical series. The incidence of IOS has likely been underestimated due to the limited use of intraoperative electrocorticography (ECoG) by neurosurgical teams, which identifies only seizures with motor characteristics, neglecting non-motor seizures [2,3].

The aim of the present study is to assess the possible role of IOS on short- and long-term epileptological and clinical outcomes in patients with TRE who underwent surgery for DLGGs, with intraoperative neurophysiological monitoring (IOM) and ECoG.

## 2. Materials and Methods

### 2.1. Study Population

This is a retrospective cohort study of 154 consecutive patients with DLGGs and TRE, who underwent surgery between 1 January 2007 and 31 May 2018. The 2017 ILAE (International League Against Epilepsy) classification was applied to classify seizures [5]. All patients were under anti-seizure medications (ASMs).

Data on pre-operative neurological status, seizure semeiology and frequency, pre-operative electroencephalographic (EEG) recordings, ASM regimen, pre-operative brain magnetic resonance imaging (MRI) findings, anesthetic protocol and intraoperative electrophysiological data, occurrence of IOS, histology, occurrence of early post-operative seizures (POS), seizure and clinical outcomes at 12 and 24 months, tumor recurrence, and mortality at 24 months were retrieved from clinical charts.

Patients were enrolled according to the following criteria:

- Age ≥ 18 years;
- Pre-operative brain MRI suggestive of supratentorial DLGGs;
- No previous surgery;
- No pre-operative chemo- or radiotherapy;
- Objective evaluation of the extent of resection (EOR) on MRI images in DICOM format based on T2-weighted MRI sequences;
- Retrospective review of neurophysiological data (EEGs and ECoGs);
- Neurological follow-up at our Epilepsy Center (Udine, Italy).

We considered early POS as all seizures occurring within seven days after surgery [6]. 

The present study was approved by the local ethics committee, Comitato Etico Unico Regionale del Friuli Venezia Giulia (protocol N.0036567/P/GEN/EGAS, ID study 2540). Written informed consent was obtained for surgery. Considering that the study was retrospective, written consent to participate in the study was not applicable (or not obtained or not needed).

### 2.2. Pre-Operative EEG Recording

Each patient underwent a pre-operative EEG recording (32-channel EB Neuro Mizar Sirius system with Galileo NT software, EB Neuro, (https://www.ebneuro.com/en/, accessed on 24 April 2025)), according to the 10–20 International System. EEGs were scored as “normal”, “slow”, or “epileptic”, as described in a previous paper [3].

### 2.3. Anesthetic Protocol

Total intravenous anesthesia (TIVA) with propofol and remifentanil infusions was used for patients operated on under general anesthesia.

Awake surgery was selected in all cases with lesions of the dominant hemisphere, following the standard protocol in use at our institution [7]. In the case of awake craniotomy, remifentanil was used at a median dose of 0.02 µg/Kg/min. The scalp was injected with local anesthetic (20 mL 2% lidocaine). Low doses of propofol were allowed only at the end of surgery.

### 2.4. Intraoperative Electrocorticography and Brain Mapping

ECoGs were recorded using a 32-channel machine (Axon System Eclipse^®^) and carried out by experienced neurophysiologists. They were separately analyzed offline by two neurophysiologists (G.P. and C.L.), blinded to the patients’ outcomes.

Recordings started before resection by placing 2–3 strip electrodes over and around the lesion. During surgery, the strips were placed on the margin of the exposed area. The reference electrode was located on the forehead (Fpz).

The low-frequency filter was set at 1 Hz, and the high-frequency filter at 70 Hz. Sensitivity was between 300 and 500 µV/mm, according to the amplitude of background and epileptiform activity.

ECoG recordings were scored as “normal”, “slow”, or “epileptic”, the latter according to the presence of localized and/or diffused epileptic activity, described following the classification of Palmini et al. [3,8].

Brain mapping was performed by direct cortical stimulation, using a bipolar electrode with 5 mm spaced tips delivering a biphasic current, with a pulse frequency of 60 Hz, an amplitude from 1 to 3.5 mA, and a maximum stimulation duration of 4 s. Current intensity was determined for each subject by progressively increasing the amplitude by 0.5 mA, starting from 1 mA, until a response could be elicited without after discharges at ECoG.

Motor evoked potentials (MEPs) mapping was achieved with a train of 4–5 monopolar anodal rectangular electrical pulses of 300 µs (range: 200–500), with an interstimulus interval of 2 ms, that were delivered to the exposed cortex via electrodes strip covering the area of interest. The reference electrode was placed at Fpz. For mapping purposes, the threshold intensity was previously calculated. Muscular responses were recorded from needle electrodes, since this kind of stimulation does not generally induce tonic movement visible to the bare-eye.

### 2.5. Intraoperative Seizure and Management

IOS were defined as any seizure, either electrographic or electroclinical, observed during surgery. Seizures were classified as electrographic if any detectable clinical sign could not be witnessed; otherwise, the seizure was scored as electroclinical when clinical symptoms were visible. Spontaneous ictal activity on the ECoG was identified by evolving discharges characterized by one or more of the following patterns: rhythmic waves (in theta, delta, or alpha bands), rhythmic spiking, repetitive spike/polyspikes-waves or an electrodecremental pattern, characterized by a general attenuation of background rhythms replaced by low-voltage, high-frequency activity [3,8,9]. These patterns had to show an abrupt onset (not temporally related to electrical stimulation), a clear evolution in amplitude, frequency, and/or topography over time, and had to last at least 10 s [3,8,9]. In case of IOS, surgical activity and stimulations were stopped, and the cortical surface was promptly irrigated with Ringer’s lactate until the seizures ceased. Mapping and MEPs monitoring were avoided until patients regained speech and motor abilities, and ECoG revealed the end of the epileptic activity.

### 2.6. Histological and Molecular Analysis

All histological samples were reviewed by two expert neuropathologists according to the *WHO Classification of Tumors of the Central Nervous System*.

Histological examination, immunohistochemistry (IHC), fluorescence in situ hybridization (FISH), and analysis of the genetic status of O-methylguanine-DNA-methyltransferase (MGMT) promoter and isocitrate dehydrogenase (IDH ½) genes were performed [10].

### 2.7. Neurological Follow-Up

Patients were clinically evaluated at 1, 3, 6, 9, 12, 18, and 24 months, according to a regular schedule.

Seizure outcome, described using the Engel classification [11], was retrieved through clinical interviews and seizure diary entries. The Karnofsky Performance Status (KPS) scale and the modified Rankin Scale (mRS) were used to assess neurological outcome and disability [12,13,14].

### 2.8. Statistical Analysis

Descriptive analysis of the main features of the study population was performed using mean ± SD (standard deviation) or median and range for continuous variables, and percentages for categorical variables.

Data were tested for normal distribution using the Shapiro–Wilk test. The t-test or Mann–Whitney U-test, as appropriate, was used to compare continuous variables between groups. For categorical variables, cross-tabulations were generated, and a chi-square or Fisher’s exact test was used to compare distributions, as appropriate.

The impact of IOS or early POS on seizure outcome, evaluated as the Engel class (dichotomized as Ia or >Ia) at 12 and 24 months, was evaluated by a univariate binary logistic regression. A similar statistical analysis was performed to evaluate the impact of IOS or early POS on clinical outcome, evaluated as the KPS score (dichotomized as <80 or ≥80) and as the mRS score (dichotomized as score < 2 or ≥2), both at 12 and 24 months of follow-up. Then, a multivariate logistic regression model was built to estimate the odds ratio (OR) of the Engel class Ia, the KPS score ≥ 80, and the mRS score < 2 at 1 and 2 years.

The results are presented as odds ratios (ORs) and 95% confidence intervals.

All analyses were conducted using Jamovi (version 2.3.28) for Mac. All two-tailed statistical significance levels were set at *p* < 0.05.

## 3. Results

### 3.1. Study Population and Pre-Operative Characteristics

We included a total of 154 patients: 95 males (61.7%) and 59 females (38.3%), with a mean age of 38.90 ± 11.30 years (range: 15–73 years).

All patients experienced at least one seizure as the initial manifestation of the disease, leading to a diagnosis of TRE. Specifically, epilepsy onset was characterized by focal seizures in 59 patients (38.3%) and focal to bilateral tonic–clonic seizures in 95 patients (61.7%). Seizure types were classified as follows: motor seizures occurred in 105 patients (68.2%), while non-motor seizures were observed in 49 patients (31.8%). More specifically, among motor seizures, 86 out of 154 patients (55.8%) presented with focal to bilateral tonic–clonic seizures, whereas 21 patients (13.6%) had exclusively focal motor seizures. Non-motor seizures were further categorized as follows: focal seizures with cognitive symptoms in 13 cases (8.4%), focal sensory seizures in 18 cases (11.7%), focal seizures with autonomic symptoms in four cases (2.6%), and focal seizures with emotional symptoms in three cases (1.9%) (Table 1). The duration of epilepsy was at least one year in the majority of cases (133/154, 86.4%).

At disease onset, all patients underwent a brain MRI, which revealed an intra-axial lesion consistent with a suspected DLGG. In 57.1% of cases (88/154), the lesion was located in the left hemisphere, while in the remaining 42.9% (66/154), the right hemisphere was involved. More specifically, the most frequently involved site was the insular region (41.6%), followed by the frontal lobe (particularly the pre-rolandic area) in 33.8% of cases, the temporal lobe in 24 patients (15.6%), and the parietal lobe (post-rolandic area) in 9.1%.

All patients underwent at least one pre-operative EEG recording: in 45.5% of cases (70 patients), EEG findings were normal, whereas in the remaining 84 patients (54.6%), abnormalities were detected, including focal slow-wave abnormalities in 44 patients (28.6%) and focal epileptiform activity in 40 patients (26%).

The majority of patients (91/154, 59.1%) presented with seizures every month before starting ASM, while only 11 patients (7.1%) had seizures daily.

All patients started ASM: 127 patients (82.5%) took monotherapy with complete seizure control, whereas 27 patients (17.5%) required polytherapy with more than two ASMs, reaching the condition of drug-resistant epilepsy. Levetiracetam was the most commonly prescribed drug (58.4%, 90/154).

Table 1 summarizes the main demographic, clinical, and radiological characteristics.

### 3.2. Intraoperative and Histological Data

All patients included in the study underwent surgical treatment. Awake craniotomy was performed in 112 cases (72.7%), while the remaining 42 patients (27.3%) underwent surgery under general anesthesia. The extent of resection (EOR) ranged from a minimum of 28% to a maximum of 100%, with a mean value of 84.25 (±15.29). More than 40% (46.8%) of patients had an EOR > 90%.

IOM was performed during all surgeries; ECoG recordings revealed epileptic activity in 81 patients (52.6%) and focal slow-wave abnormalities in 26 patients (16.9%), while no abnormalities were detected in 47 patients (30.5%). Intraoperative seizures occurred in 40 patients (26%). Specifically, we detected 62 intraoperative seizures, as some patients experienced more than one event: 47 were electroclinical seizures, while 15 were electrographic seizures detected only on the ECoG.

In the immediate post-operative period (within seven days), early seizures were observed in 28 patients (18.5%).

Histopathological analysis confirmed the diagnosis of diffuse astrocytoma in 110 cases (71.4%), with 95 cases (61.7%) harboring an IDH1/2 mutation, and 15 cases (9.7%) classified as IDH wild-type. The remaining cases were oligodendrogliomas (44/154, 28.6%). Co-deletion of 1p/19q was identified in 44 cases (28.6%), whereas MGMT promoter methylation was present in 135 cases (87.7%). The mean Ki-67 proliferation index was 5.43 (±3.85) (Table 1).

In the majority of cases (90.3%), the lesions were classified as WHO grade II, while in the remaining cases, they were WHO grade III.

### 3.3. Post-Operative Seizure, Functional and Oncological Outcomes

All patients completed the one-year follow-up. Data two years after surgery were available for 148 patients (96.1%), with six patients lost to follow-up.

At 12 months, 69.5% (107/154) of patients were seizure-free (the Engel class Ia). At two years, seizure freedom was confirmed in 64.4% (96/148) of patients.

Regarding functional outcomes, the KPS and the mRS scores were collected at 12 and 24 months. At one year, 87% (134/154) of patients had a KPS score of ≥80, which was maintained in 81.1% (120/148) at two years. The mRS score was <2 in 79.2% (122/154) of patients in the first year after surgery and remained below this threshold in 71.6% (106/148) at two years.

Tumor recurrence was observed in 88 patients (59.9%) within the two-year follow-up period. Overall, 27.7% (41/148) of patients died during the observation period, due to either tumor-related or other causes.

At univariate analysis, demographic characteristics (specifically, gender and age), tumor side and region, pre-operative epilepsy characteristics (seizure types, pharmaco-resistance, duration), pre-operative EEG features, and histopathological characteristics were not found to be associated with an increased risk of developing IOS (Table 2a). The presence of an ECoG pattern characterized by epileptiform abnormalities and the type of anesthesiologic protocol (awake surgery) were variables statistically associated with an increased risk of developing IOS (*p* = < 0.001; *p* = 0.004, respectively), confirmed also at multivariate analysis (*p* = < 0.001; *p* = 0.004, respectively) (Table 2a).

Regarding early POS, prognostic factors of an increased risk were as follows: tumor location (in particular the insular region, *p* = 0.036), presence of epileptic activity on pre-operative EEG (*p* = 0.023), and on ECoG (*p* = < 0.001) (Table 2b). In the multivariate analysis, only the presence of epileptiform activity on ECoG (*p* = 0.004) was confirmed as a prognostic factor for early POS. The occurrence of IOS did not represent a risk factor for the development of early POS (*p* = 0.892) (Table 2b).

The potential prognostic role of both IOS and early POS was also evaluated in terms of long-term epileptological, functional, and oncological outcomes.

The occurrence of IOS did not significantly affect post-operative seizure control, assessed using the Engel classification, at either 12 months (*p* = 0.403) or 24 months (*p* = 0.488), independently of pre-operative epilepsy control (Table 3a). The most relevant prognostic factor for seizure outcome was the EOR at 12 and 24 months (*p* = 0.027; *p* = 0.048) (Table 3a). Similarly, no significant correlation was found between IOS and the KPS or mRS scores at 12 months (*p* = 0.748; *p* = 0.363, respectively) and 24 months (*p* = 0.975; *p* = 0.538, respectively) (Table 3b,c). No correlation was observed between IOS occurrence and increased risk of tumor recurrence (*p* = 0.561) or mortality at two years (*p* = 0.930).

In contrast, in the univariate analysis, a statistically significant correlation was identified between the occurrence of early POS and the Engel classification at 12 and 24 months (*p* = < 0.001; *p* = < 0.001), confirmed in the multivariate analysis at 12 months (*p* = 0.005), but not at 24 months (*p* = 0.102) (Table 3a). In the univariate analysis, the development of early POS showed worse KPS and mRS scores at 12 months (*p* = 0.034; *p* = 0.009) and 24 months (*p* = 0.032; =0.003). Furthermore, the multivariate analysis did not confirm these statistical correlations, both for the KPS and mRS scores (*p* = 0.324 and *p* = 0.168 at 12 months; *p* = 0.920 and *p* = 0.165 at 24 months) (Table 3b,c). However, no significant correlation was found between early POS and the risk of tumor recurrence (*p* = 0.563) or mortality (*p* = 0.438).

Finally, the relationship between seizure control and functional outcome was assessed. Complete seizure control (the Engel class Ia) was statistically correlated with a higher KPS score at 24 months in the univariate analysis (*p* = 0.010) (Table 3a). The same was found for the mRS score both at 12 and 24 months (*p* = < 0.001). The multivariate analysis showed the same significant correlation only for seizure control and the mRS score at 2 years (*p* = 0.041) (Table 3). Moreover, poor seizure control (the Engel class > Ia) at 24 months was statistically correlated with a higher risk of tumor recurrence (*p* = 0.007) and mortality at two years (*p* = 0.030).

## 4. Discussion

Intraoperative seizures represent a complication of neurosurgical procedures, particularly those performed on awake patients [1,15]. The literature focuses on risk factors and management of IOS, while data are lacking regarding their possible post-operative consequences, such as impact on early POS or long-term seizure and functional outcomes.

Our data indicate that the occurrence of IOS does not affect either long-term seizure control, assessed by the Engel class at 12 and 24 months, or functional outcomes assessed by the KPS and mRS scores. Furthermore, the risk of tumor recurrence and mortality also appears unaffected by the development of IOS.

To our knowledge, this is the first study specifically addressing the long-term prognostic implications of IOS in patients with TRE, highlighting their lack of impact on seizure and functional outcomes, in contrast to the role played by early POS.

IOS induced by cortical or subcortical stimulation and surgical manipulation can be considered as acute symptomatic seizures. Acute symptomatic seizures are etiologically linked to a specific and limited triggering cause, such as a documented brain insult or systemic pathological conditions (intoxications, infections, metabolic disarrangement, etc.), to which the seizures can be temporally correlated [6]. In this context, an acute symptomatic seizure represents a symptom of a transient disturbance in cerebral activity, which does not lead to alterations in the neuronal network that would favor the development or exacerbation of epilepsy [16].

In cases of spontaneous IOS, likely expressions of pre-existing epilepsy or reduced seizure threshold, their prompt recognition and treatment can prevent the onset of seizure clusters or secondary generalization during surgery. However, even spontaneous IOS did not represent risk factors for POS, and they did not worsen post-operative seizure and clinical outcomes. In fact, various authors suggested that IOS are not predicted by the risk factors for TRE reported in the literature, thus reinforcing the hypothesis that IOS has a different pathogenesis and a different propagation [17,18,19,20].

In our study, IOS occurrence was not statistically related to an increasing risk of early POS. This result is consistent with that of Abecassis et al. [21]. In their retrospective study on 229 consecutive patients who underwent surgery for DLGGs with ECoG monitoring, they did not find correlations between the occurrence of IOS and early POS.

The presence of epileptic activity on ECoG was found to be the only factor correlated with an increased risk of developing IOS and early POS in our sample, underscoring the close relationship between the pre-existing establishment of epileptic networks, increased cortical excitability and susceptibility to seizure development under stress conditions. Our data are in contrast with the work of Nossek et al., who did not observe any relationship between the epileptic activity detected by ECoG and the incidence of IOS [1].

Specifically, in our cohort, we found that the presence of epileptiform activity on intraoperative ECoG (ioECoG) was significantly associated with both IOS and early POS, suggesting a possible role of ioECoG as a biomarker of cortical excitability. IoECoG has long been used to guide resections in epilepsy surgery, aiming to minimize the risk of leaving epileptogenic tissue behind [22,23]. It can be used in various scenarios, particularly to identify epileptically active cortical areas adjacent to a lesion targeted for resection. This allows surgeons to extend resections to include tissue potentially involved in the seizure onset, thereby improving epileptological outcomes. However, the interpretation of ioECoG findings can be challenging, limiting the reliability of interictal epileptiform discharges (IEDs) detection for surgical decision-making [22,24,25]. Despite its widespread use, the prognostic value of ioECoG in TRE remains controversial. Favorable seizure outcome (FSO) after ioECoG-tailored resection is highly variable in the literature, with most evidence deriving almost entirely from retrospective observational studies. In a recent meta-analysis, Guo et al. [23] reported an overall pooled FSO rate of 74% after epilepsy surgery with ioECoG, particularly in tumor-related cases. Better outcomes were observed in studies comparing tailored vs. non-tailored procedures, and in cases where IED areas were completely resected [23]. To better clarify the clinical utility of ioECoG in TRE, further research is warranted. Both retrospective and prospective cohort studies comparing surgeries with and without ioECoG guidance are needed. Such studies should include detailed information on the underlying pathology, MRI findings, surgical location, and final seizure outcomes.

Early POS, which occurs within seven days following a surgical procedure, manifests in 3% to 18% of patients with brain tumors who do not have a history of epilepsy, while the incidence is higher in patients who present with pre-operative seizures [26].

In our study, a correlation was identified between the development of early POS and both epileptological and functional outcomes. In contrast, no correlation was found between early seizures and tumor recurrence or mortality. These results may be attributed to the fact that early seizures can lead to intracranial hemorrhages, cerebral hypoxia, or intracranial hypertension, potentially resulting in the emergence or exacerbation of neurological deficits. Furthermore, various studies have observed that the length of hospitalization following surgery is greater in patients who experience early POS; specifically, the hospitalization length increases from an average of 8.9 days to 14.4 days [2,27].

Finally, the occurrence of status epilepticus (SE) in the post-operative period is associated with increased morbidity and mortality, particularly when it is a non-convulsive SE. Adverse epileptological outcomes may be explained by the emergence of new cerebral insults induced by early seizures, as well as the persistence of the epileptogenic focus. For this reason, the possibility of evaluating ECoG activity and detecting any electrical or non-motor seizures ensures an appropriate adjustment of ASM in the immediate post-operative period and allows for more accurate and attentive EEG monitoring.

The study by Marku et al. [28] demonstrated that among 3763 patients investigated with a diagnosis of grade II, III, and IV glioma, there was an increase in mortality among patients who experienced seizures in the post-operative period [28]. Specifically, patients with pre-operative epilepsy exhibited the highest mortality rates, regardless of the WHO grade. In contrast, in the cohort with de novo epilepsy, mortality was found to be independent of both the tumor grade and the type of treatment undertaken.

Another study indicated that post-operative epilepsy does not lead to a reduction in survival, except when seizures develop more than eight months post-surgery. In our study, the absence of correlation between the development of early POS and patient mortality is related to the nature of our sample, which consisted mainly of patients with grade II gliomas; thus, if treated with maximal surgery, they tend to have longer survival [29]. Furthermore, the majority of our patients were seizure-free during the follow-up, and the literature emphasizes that freedom from seizures is associated with a lower risk of progression to more aggressive tumor forms [30,31].

The work of Pallud et al. [32] demonstrated both improved outcomes in patients with high-grade brain tumors treated with levetiracetam and an in vitro anti-oncogenic role of commonly used ASMs in patients with gliomas [32]. Moreover, clinical studies on perampanel, a drug that acts as a non-competitive AMPA receptor antagonist, exhibited promising results regarding its anti-tumor effects [33,34]. These data suggest that gliomas and epilepsy share numerous pathogenetic pathways, and patients may benefit from a direct or indirect role of ASMs in potential oncological mechanisms, through the modulation of interictal epileptic activity [35].

Epileptic networks in subjects with brain tumors are the result of complex and various pathogenetic mechanisms encompassing alterations in neuronal excitability, abnormal neural network activity, and changes in the tumor microenvironment. Genetic mutations and associated molecular pathways are crucial factors sustaining both brain tumor development and epilepsy [36]. Isocitrate dehydrogenase mutations, causing accumulation of D-2-hydroxiglutarate (D2-HG) in tumors, are responsible for increased glutamatergic activity and seizure susceptibility [37,38]. In addition to glutamatergic dysfunction, which also promotes glioma cell proliferation, evidence supports defective inhibitory signaling in TRE, including changes in the expression of neuronal chloride co-transporters, resulting in alterations of chloride homeostasis and GABA-induced depolarization [39].

Moreover, intra- and peri-tumoral inflammatory response, mediated by astrocytes and microglia, may contribute to disrupting the excitatory–inhibitory balance [40].

Experimental and clinical studies support the theory of bidirectional interactions between neurons and glioma cells: neuronal epileptiform activity sustains glioma cells’ proliferation, growth, and mobility, and vice versa [41,42].

Finally, seizure-free patients have higher KPS and lower mRS scores because seizures significantly impact their quality of life and increase the subjective perception of being ill [43].

The persistence of seizures, regardless of any residual tumor, is associated with a risk of morbidity and mortality linked to accidents, falls, trauma, and hematomas. Seizures may complicate oncological management, particularly when they are frequent and exhibit generalized features (epileptic falling and secondary tonic–clonic generalization) [43].

### Limitations and Strengths

The primary limitation of this study is its retrospective nature, which is associated with inherent biases characteristic of such study designs. Additionally, because the studied population spans an extensive timeframe, there have been advancements in neurosurgical, anesthetic, neurophysiological, and anatomical–pathological techniques, making the series not completely homogeneous. However, the monocentric nature of the study ensures uniform standards over time; furthermore, the selected patients are homogeneous concerning the type of tumor and history of TRE. Finally, all patients were neurologically evaluated by epileptologists with extensive experience in this field and underwent IOM with ECoG recordings.

## 5. Conclusions

Intraoperative seizures represent a recognized complication of neurosurgical procedures, posing various challenges during surgery. Notwithstanding, there is a lack of literature regarding their effects in the early post-operative period and in the long term.

Our study suggests that IOS are not correlated with functional and seizure outcomes, mortality, and tumor recurrence. In contrast, early POS negatively impact both seizure control and functional outcomes.

IOS are comparable to acute symptomatic seizures and therefore do not lead to medium- and long-term complications. Conversely, early POS and the persistence of epilepsy even after surgical resection are associated with poorer epileptological and functional outcomes.

In this context, intraoperative ECoG emerges as a valuable tool, not only for optimizing the EOR but also for identifying subclinical IOS and patients at higher risk of early POS. Its predictive role may assist in stratifying patients who could benefit from enhanced surveillance and tailored anti-seizure treatment. Future prospective studies are needed to validate the prognostic value of ECoG and determine whether ioECoG-guided resections can improve long-term outcomes in patients with TRE.

Ultimately, optimizing therapy in both the pre-operative and post-operative periods, along with careful clinical and instrumental follow-up of operated patients, remains crucial for improving overall management and clinical outcomes in patients with brain tumors and epilepsy.

## Figures and Tables

**Table 1:** Baseline characteristics of the study population.

Variables	
No. of patients	154
**Sex, n (%)**	
Male	95 (61.7)
Female	59 (38.3)
**Age, (years)**	
Mean (±DS)	38.90 (±11.30)
Range	15–73
**Seizure Onset**	
Focal seizures	59 (38.3)
Focal to bilateral tonic-clonic seizures	95 (61.7)
**Seizure Types**	
Motor	105 (68.2)
Non-motor	49 (31.8)
Autonomic	4 (2.6)
Cognitive	13 (8.4)
Emotional	3 (1.9)
Sensory	18 (11.7)
**Pre-operative seizures frequency**	
Monthly	91 (59.1)
Weekly	52 (33.8)
Daily	11 (7.1)
**ASM regimen**	
Monotherapy	127 (82.5)
Polytherapy	27 (17.5)
**Pre-operative EEG features**	
Normal	70 (45.5)
Slow	44 (28.6)
Epileptic	40 (26)
**Tumor side**	
Left	88 (57.1)
Right	66 (42.9)
**Tumor site**	
Frontal	52 (33.8)
Parietal	14 (9.1)
Temporal	24 (15.6)
Insular	64 (41.6)
**Pre-operative tumor volume (T2-w MRI images-cm^3^)**	
Mean (±DS)	55.28 (±38.19)
Range	6–250
**EOR %** (range)	84.25 ± 15.29 (28–100)
**Molecular Class**	
Oligodendroglioma IDH1/2 mutated 1p-19q codeleted	44 (28.6)
Diffuse astrocytoma IDH1/2 mutated	95 (61.7)
Diffuse astrocytoma IDH1/2 wild-type	15 (9.7)
**MGMT promoter methylation**	
Yes	135 (87.7)
No	19 (12.3)
**WHO grade**	
Grade II	139 (90.3)
Grade III	15 (9.7)
**Anesthesiologic Protocol**	
Awake surgery	112 (72.7)
General anesthesia	42 (27.3)
**Intraoperative ECoG features**	
Normal	47 (30.5)
Slow	26 (16.9)
Epileptic	81 (52.6)
**Intraoperative seizures (IOS)**	
Yes	40 (26)
No	114 (74)
**Early post-operative seizures (POS)**	
Yes	28 (18.5)
No	123 (81.5)

Patients’ characteristics are described using mean and range for continuous variables, and the number of cases with relative percentage (in parentheses) for categorical variables.

**Table 2:** (**a**) Predictors of IOS development. (**b**) Predictors of early POS development.

(a)										
	IOS		Univariate Analysis			IOS		Multivariate Analysis		
	NO	YES	O.R.	CI (95%)	*p*	NO	YES	O.R.	CI (95%)	*p*
**Variables**	**114**	**40**								
**Sex, n (%)**										
*Male*	70 (61.4)	25 (62.5)	0.955	0.454–2.007	0.902					
*Female*	44 (38.6)	15 (37.5)								
**Age (mean ± DS)**	38.07 ± 10.18	41.88 ± 14.76	1.008	0.969–1.049	0.677					
**Tumor site, n (%)**										
*Frontal (pre-central)*	33 (28.9)	19 (47.5)								
*Parietal (post-central)*	12 (10.6)	2 (5)	1.607	0.617–4.184	0.332					
*Temporal*	18 (15.8)	6 (15)								
*Insular*	51 (44.7)	13 (32.5)								
**Pre-operative epilepsy features**										
**Seizure type, n (%)**										
*Motor*	73 (64)	32 (80)	0.445	0.188–1.056	0.062					
*Non motor*	41 (36)	8 (20)								
**Seizure Onset, n (%)**										
*Focal*	47 (41.2)	12 (30)	1.637	0.756–3.543	0.209					
*Focal to bilateral tonic clonic*	67 (58.8)	28 (70)								
**ASM regimen, n (%)**										
*Monotherapy*	94 (82.5)	33 (82.5)	0.997	0.386–2.572	0.995					
*Polytherapy*	20 (17.5)	7 (17.5)								
**Duration, n (%)**										
≥*1 year*	97 (8.51)	36 (90)	1.577	0.497–5.003	0.436					
*<1 year*	17 (14.9)	4 (10)								
**EOR (mean ± DS)**	84.33 (±15.14)	84.00 (±15.92)	1.001	0.978–1.025	0.905					
**Pre-operative EEG, n (%)**										
*Not epileptiform*	84 (73.7)	31 (77.5)	0.813	0.347–1.904	0.633					
*Epileptiform*	30 (26.3)	9 (22.5)								
**EcoG, n (%)**										
*Not Epileptiform*	64 (56.1)	9 (22.5)	4.409	1.924–10.103	**<0.001**			6.076	2.250–16.409	**<0.001**
*Epileptiform*	50 (43.9)	31 (77.5)								
(**b**)										
	**Early POS**		**Univariate Analysis**			**Early POS**		**Multivariate Analysis**		
	**NO**	**YES**	**O.R.**	**CI (95%)**	***p***	**NO**	**YES**	**O.R.**	**CI (95%)**	***p***
**Variables**	**123**	**28**								
**Sex, n (%)**										
*Male*	76 (68.3)	17 (60.7)	1.046	0.451–2.426	0.916					
*Female*	47 (37.3)	11 (39.3)								
**Age (mean ± DS)**	38.07 ± 10.18	41.88 ± 14.76	1.072	0.391–2.937	0.338					
**Tumor site, n (%)**										
*Frontal (pre-central)*	45 (36.6)	6 (21.4)	1.706	0.617–4.184	**0.036**			5.177	0.950–28.206	0.06
*Parietal (post-central)*	10 (8.1)	4 (14.3)								
*Temporal*	22 (17.9)	1 (3.6)								
*Insular*	46 (37.4)	17 (60.7)								
**Pre-operative epilepsy features**										
**Seizure type, n (%)**										
*Motor*	87 (70.7)	16 (51.1)	1.813	0.780–4.212	0.163					
*Non motor*	36 (29.3)	12 (42.9)								
**ASM regimen, n (%)**										
*Monotherapy*	101 (82.1)	23 (82.1)	0.998	0.342–2.914	0.997					
*Polytherapy*	22 (17.9)	5 (17.9)								
**Duration, n (%)**										
≥*1 year*	109 (88.6)	21 (75)	0.385	0.139–1.069	0.060					
*<1 year*	14 (11.4)	7 (25)								
**EOR (mean ± DS)**	83.93 (±15.98)	84.86 (±12.48)	0.996	0.969–1.024	0.772					
**Pre-operative EEG, n (%)**										
*Not epileptiform*	96 (78)	16 (57.1)	2.667	1.127–6.312	**0.023**			2.009	0.685–5.897	0.204
*Epileptiform*	27 (22)	12 (42.9)								
**EcoG, n (%)**										
*Not Epileptiform*	66 (53.6)	5 (17.8)	5.326	1.902–14.919	**<0.001**			6.005	1.754–20.560	**0.004**
*Epileptiform*	57 (46.4)	23 (82.2)								
**Intraoperative seizures, n (%)**										
*Yes* *No*	32 (26) 91 (74)	8 (28.6) 20 (71.4)	1.137	0.456–2836	0.782					

Statistically significant results are in bold.

**Table 3:** (**a**) Seizure outcomes at 12 and 24 months. (**b**) Functional outcomes assessed using the KPS score at 12 and 24 months. (**c**) Functional outcomes assessed using the mRS score at 12 and 24 months.

(a)						
	Seizure Outcome at 12 Months			Seizure Outcome at 24 Months		
	O.R.	CI (95%)	*p*	O.R.	CI (95%)	*p*
**Variables**						
**Age**	1.042	0.995–1.092	0.078	1.045	0.003–5.662	**0.047**
**Seizure type** (*motor*)	4.709	1.548–14.321	**0.007**	1.048	0.381–2.886	0.928
**ASM regimen** (*polytherapy*)	5.278	1.539–18.108	**0.008**	1.042	0.319–3.407	0.945
**Epilepsy Duration** (<*1 year*)	0.668	0.162–2.750	0.577	1.142	0.310–4.205	0.842
**Pre-operative EEG** (*epileptiform*)	1.258	0.430–3.682	0.675	1.329	0.498–3.548	0.570
**EcoG** (*epileptiform*)	5.099	1.681–15.464	**0.004**	4.615	1.675–12.709	**0.003**
**EOR**	0.967	0.939–0.996	**0.027**	0.973	0.946–1.000	**0.048**
**WHO grade** (*II vs. III*)	1.548	0.262–9.145	0.630	1.606	0.284–9.074	0.592
**IOS** (*yes*)	0.636	0.220–1.837	0.403	1.428	0.521–3.917	0.488
**Early POS** *(yes)*	5.681	1.673–19.283	**0.005**	0.408	0.139–1.195	0.102
**KPS** (*≥80*) **at 1 y and 2 y**	0.471	0.074–3.010	0.426	1.792	0.420–7.655	0.431
**mRS** (*<2*) **at 1 y and 2 y**	2.072	0.506–8.486	0.311	3.584	1.051–12.224	**0.041**
(**b**)						
	**Functional Outcome (KPS) at 12 Months**			**Functional Outcome (KPS) at 24 Months**		
	**O.R.**	**CI (95%)**	***p***	**O.R.**	**CI (95%)**	***p***
**Variables**						
**Age**	1.058	0.9986–1.136	0.117	1.044	0.977–1.116	0.201
**Seizure type** (*motor*)	0.452	0.068–3.009	0.412	0.398	0.068–2.333	0.307
**ASM regimen** (*polytherapy*)	1.103	0.151–8.044	0.923	1.126	0.187–6.784	0.897
**Epilepsy Duration** (<*1 year)*	7.036	0.823–60.158	0.075	3.984	0.471–33.699	0.204
**EOR**	1.032	0.974–1.0.93	0.287	1.006	0.960–1.054	0.797
**WHO grade** (*II vs. III*)	3.722	0.629–22.038	0.181	0.594	0.035–10.141	0.719
**IOS** (*yes*)	0.749	0.128–4.371	0.748	1.025	0.214–4.922	0.975
**Early POS** (*yes*)	0.380	0.056–2.595	0.324	0.924	0.196–4.349	0.920
**Engel class** (*Ia*) **at 1 y and 2 y**	0.612	0.103–3.619	0.588	1.553	0.361–6.684	0.554
**mRS** (*<2*) **at 1 y and 2 y**	122.751	16.756–899.274	**<0.001**	198.926	21.248–1862.359	**<0.001**
(**c**)						
	**Functional Outcome (mRS) at 12 Months**			**Functional Outcome (mRS) at 24 Months**		
	**O.R.**	**CI (95%)**	***p***	**O.R.**	**CI (95%)**	***p***
**Variables**						
**Age**	0.997	0.946–1.051	0.916	0.978	0.920–1.039	0.475
**Seizure type** (*motor*)	0.893	0.254–3.142	0.861	0.827	0.225–3.044	0.775
**ASM regimen** (*polytherapy*)	1.089	0.202–5.874	0.921	0.896	0.169–4.758	0.897
**Epilepsy Duration** (<*1 year*)	0.366	0.058–2.296	0.283	0.427	0.063–2.903	0.384
**EOR**	0.977	0.941–1.013	0.210	0.980	0.942–1.019	0.315
**WHO grade** (*II vs. III*)	0.879	0.107–7.253	0.905	1.514	0.134–17.174	0.738
**IOS** (*yes*)	1.907	0.474–7.668	0.363	1.520	0.402–5.752	0.538
**Early POS** (*yes*)	0.378	0.095–1.504	0.168	0.384	0.099–1.485	0.165
**Engel class** (*Ia*) **at 1 y and 2 y**	2.282	0.599–8.696	0.227	3.367	0.990–11.449	0.052
**KPS** (*≥80)* **at 1 y and 2 y**	94.976	14.860–607.044	**<0.001**	202.139	20.384–2004.501	**<0.001**

Statistically significant results are in bold.

## Data Availability

The raw data supporting the conclusions of this study are not openly available due to reasons of sensitivity and are available from the corresponding author upon reasonable request.

## Abbreviations

The following abbreviations are used in this manuscript:

ASMs	anti-seizure medications
CNS	central nervous system
DLGGs	diffuse low-grade gliomas
ECoG	electrocorticography
EEG	electroencephalography
EOR	extent of resection
FISH	fluorescence in situ hybridization
FSOKPS	favorable seizure outcomeKarnofsky Performance Status
IDH	isocitrate dehydrogenase
ILAE	International League Against Epilepsy
IHC	immunohistochemistry
IoECoGIOM	Intraoperative electrocorticographyintraoperative neurophysiological monitoring
IOS	intraoperative seizures
MEP	motor evoked potential
MGMT	O6-methylguanine-DNA-methyltransferase
MRI	magnetic resonance imaging
mRS	modified Rankin Scale
OR	odds ratio
POS	post-operative seizures
SE	status epilepticus
TIVA	total intravenous anesthesia
TRE	tumor-related epilepsy
WHO	World Health Organization
